# Microbial Community Characteristics and Underlying Drivers Along the Streams, Tributaries, and Main Stems of the Yangtze River Source Region

**DOI:** 10.1002/ece3.71290

**Published:** 2025-04-15

**Authors:** Futing Liu, Luyao Kang, Lele Lin, Sisi Yu

**Affiliations:** ^1^ Key Laboratory of Forest Ecology and Environment of National Forestry and Grassland Administration, Ecology and Nature Conservation Institute Chinese Academy of Forestry Beijing China; ^2^ State Key Laboratory of Vegetation and Environmental Change, Institute of Botany Chinese Academy of Sciences Beijing China

**Keywords:** 16S amplicon sequencing, alpha diversity, bacterial community composition, beta diversity, Yangtze River

## Abstract

Microorganisms are regarded as the key driver in regulating biogeochemical cycles. A comprehensive examination of the riverine microbial community composition and structure is crucial for understanding the functions of aquatic ecosystems. However, previous studies mainly focused on the spatial distribution of microorganisms in the main stems of rivers, ignoring the shifts in microbial communities along the streams, tributaries, and main stems of rivers. To address this gap, we collected water samples from 27 sites along the Yangtze River Source Region covering a 1000 km reach. Based on 16S amplicon sequencing, spectral and physicochemical analyses, we examined the microbial communities as well as their influencing factors along the stream orders. Our results revealed that the bacterial community composition in streams and tributaries was different from that in the main stems of rivers. Although no significant changes were observed in bacterial alpha diversity, the structure of bacterial communities significantly changed and their beta diversity increased along the streams, tributaries, and main stems of rivers, indicating the enhanced complexity of bacterial communities. This observation was further confirmed by network analysis revealing the increased links between bacterial communities along the stream orders. However, the predicted functional potential of bacteria engaged in carbon and nitrogen cycling gradually decreased from tributaries to main stems of rivers, illustrating a higher elemental cycling and associated ecological process in the headwater streams. Based on multiple statistical analyses, we found that the dominant driver affecting riverine bacterial communities was the environment, rather than substrate and nutrient variables. Moreover, deterministic processes exerted a dominant role in regulating bacterial community assembly, despite the increased relative contribution of stochastic processes in structuring the bacterial communities along the stream orders. This study revealed the variations of bacteria and their drivers from the perspective of stream orders, providing a new understanding of the microbial communities in high‐altitude rivers.

## Graphical abstract

Bacterial beta diversity increased along the streams, tributaries, and main stems of the Yangtze River Source Region despite no significant changes in alpha diversity. The functional potentials associated with carbon and nitrogen cycling decreased from tributaries to main stems of rivers. The dominant driver affecting riverine bacterial communities was the environment rather than substrate and nutrient variables.

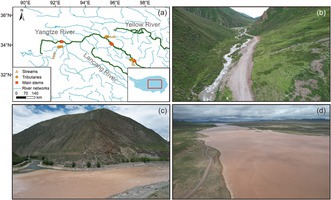

## 1 Introduction

Microorganisms are one of the key factors in driving biogeochemical cycles (Falkowski et al. 2008; Crowther et al. 2019). On the one hand, microorganisms play an important role in the riverine material cycles and consequently impact fluvial environments (Gao et al. 2017; Roberto et al. 2018). On the other hand, microbial communities could also be altered by the changes in riverine environments, which in turn affect microbially mediated ecological processes, such as greenhouse gas emissions, carbon sequestration, and related element (carbon and nitrogen) cycling (Liu et al. 2018; Zhang et al. 2019; Overholt et al. 2022). Therefore, it is necessary to explore the riverine microbial community composition and structure to gain a comprehensive understanding of aquatic ecosystem functions.

Although substantial studies have been conducted on the composition and diversity of microbial communities in rivers (Garcia et al. 2013; Hauptmann et al. 2016; Toyama et al. 2017; Raso et al. 2023), there remain two deficiencies in the following aspects. First, previous studies mainly concentrated on microorganisms in water bodies from non‐permafrost areas (Toyama et al. 2017; Raso et al. 2023), while the examination of microorganisms in rivers affected by permafrost was insufficient, especially in high‐altitude rivers. Compared with fluvial ecosystems in low‐altitude areas, both water temperature and nutrient concentrations (including inorganic nitrogen and phosphorus) are lower in high‐altitude rivers, which are also exposed to stronger ultraviolet radiation (Zhang et al. 2019). Thus, the composition and structure of microbial communities in high‐altitude rivers may be different from those in low‐altitude areas without permafrost effect. However, the examination of microbial communities in high‐altitude rivers remains inadequate to date.

Second, previous studies mainly focused on the spatial distribution of microbial communities in the main stems of rivers (Toyama et al. 2017; Liu et al. 2018; Zhang et al. 2019; Raso et al. 2023), with a limited understanding of the changes in microbial composition and diversity along the stream orders, including streams, tributaries, and main stems of rivers. Given that environments, substrates, and nutrients may be different between these three levels of stream orders (Qu et al. 2018), the composition and structure of microbial communities could also be distinct, which consequently impacts microbially mediated biogeochemical cycles within riverine ecosystems. However, it has been reported that the biogeographic pattern of bacterial communities in freshwater networks may be influenced by the upstream freshwater (Hauptmann et al. 2016) and terrestrial microorganisms (Ruiz‐González et al. 2015). Hence, more studies are needed to explore whether and how stream orders affect the variations of microorganisms in rivers.

The Tibetan Plateau, the “water tower of Asia”, is the source of the Yangtze River (Liu et al. 2018). Due to the high altitude of the plateau, rivers in this region are affected by permafrost, and the proportion of old carbon in water bodies is even higher than that of Arctic rivers (Qu et al. 2017). Coupled with the dense water network and large terrain fluctuations, the plateau provides an ideal platform to explore microbial dynamics from streams, tributaries, and main stems of rivers affected by frozen soil. In this study, we sampled waters from 27 locations along the Yangtze River Source Region covering a 1000 km reach and then determined the bacterial communities and their underlying drivers. We sought to test the following three hypotheses: (1) Due to the large differences in environments, nutrients, and substrates from the three levels of stream orders in the Yangtze River (Qu et al. 2018), the bacterial composition and community structure would also be distinct; (2) Compared with nutrients and substrates, environment variables may be the dominant factor shaping the bacterial communities; (3) Deterministic processes rather than stochastic processes dominantly regulate the bacterial community assembly due to the harsh environment of the river.

## 2 Materials and Methods

### 2.1 Study Site

**FIGURE 1:**
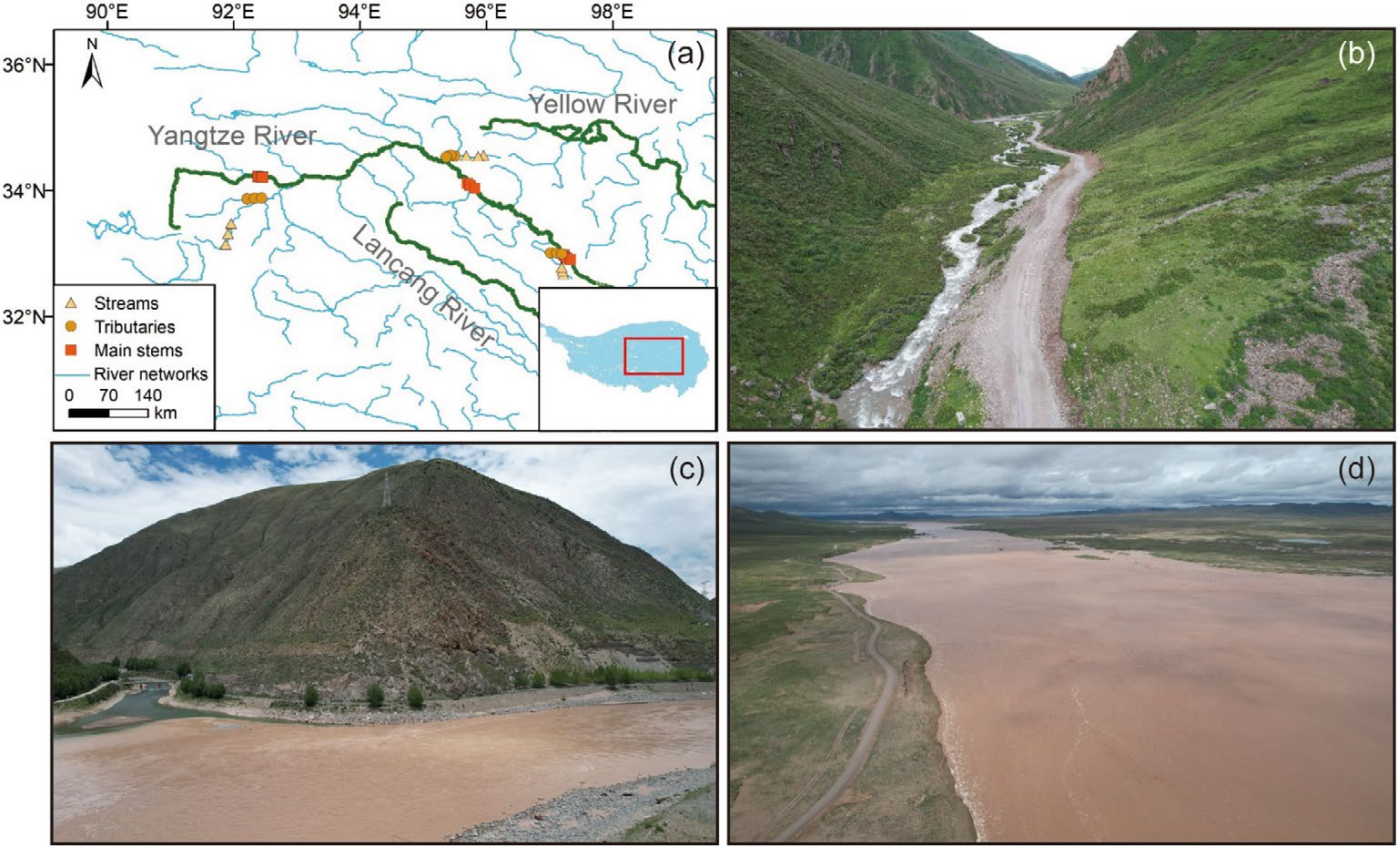
Sampling sites and representative landscapes of the Yangtze River Source Region. (a) Sampling location in the upper reaches of Yangtze River. The red square box indicates the specific location of sampling sites on the Tibetan Plateau. (b–d) Individual features of streams, tributaries, and main stems of the rivers (photo credit for panels b–d: F.T. Liu).

The research area is located in the central part of the Tibetan Plateau (Figure 1), belonging to the upper reaches of the Yangtze River with an altitude between 3455 and 4838 m (Table S1). The annual average temperature ranges from −4.2°C to 2.9°C, and the mean annual precipitation is between 264 and 487 mm. The vegetation type is alpine grassland and alpine meadow (Editorial Committee for Vegetation Map of China 2001) underlain with permafrost and seasonally frozen ground (Zou et al. 2017), mainly consisting of *Stipa purpurea*, *Carex moorcroftii*, *Kobresia pygmae*, and *Kobresia humilis*, respectively (Zhang et al. 1988). In addition, according to the World Reference Base for Soil Resource, the soil type within this area is Cambisol (IUSS Working Group WRB 2014).

### 2.2 Water Sampling

To determine the microbial communities in rivers, we collected water samples from the Yangtze River Source Region on the Tibetan Plateau during July and August in 2023 (Figure 1). Specifically, three sampling areas, including Ulran Moron, Qumarlêb, and Yushu sampling zones, were selected in the plateau with a distance of ~1000 km (Table S1). In each area, water samples were collected along the stream orders including streams (*n* = 3), tributaries (*n* = 3), and main stems (*n* = 3). Three sampling sites (with an interval of 15–30 km) were set up for each stream order. In total, we selected 27 sites, in which 27 surface water samples (sampling depth = 20 cm, volume = 2 L) were acquired across the sampling areas. Subsequently, all the collected samples were divided into two parts: one part was filtered through a 0.22 μm filter membrane (Sterivex GP 0.22, Millipore) in the field, and the filter membranes were then gathered for microbial DNA extraction and sequencing analysis. The other part was filtered through a 0.7 μm (GF/F, Whatman) membrane, and the filtered water was collected for physicochemical and spectral analyses.

### 2.3 DNA Extraction and High‐Throughput Sequencing

To clarify the characteristics of microbial communities along the streams, tributaries, and main stems of rivers, we extracted microbial DNA from the water samples and performed 16S amplicon sequencing. First, microbial DNA from a 0.22 μm filter membrane was extracted using the Power Water DNA Isolation Kit (MoBio Laboratories Inc., Carlsbad, USA). The concentration and quality of the extracted DNA were then determined using the NanoDrop‐2000 (Thermo Fisher Scientific Inc., Madison, USA). Second, the primer pair 515F/806R (5′‐GTGCCAGCMGCCGCGGTAA‐3′/5′‐GGACTACVSGGGTATCTAAT‐3′) was applied to amplify the V4 region of the 16S rRNA gene (Chen et al. 2021). The PCR reaction parameters were set as follows: 94°C for 3 min, 35 cycles of 94°C for 45 s, 50°C for 60 s, and 72°C for 60 s, and finally extended at 72°C for 10 min. The obtained PCR products were purified using the QIAEX II Gel Extraction Kit (Qiagen GmbH; Hilden, Germany) and quantified with the Quant‐iT dsDNA HS Assay Kit (Invitrogen, Carlsbad, USA). Finally, the paired‐end reads (2 × 250‐bp) were performed using the Illumina MiSeq machine (Illumina Inc., Santiago, USA).

We used the Magigene Cloud Platform (a common platform developed by Guangdong Magigene Technology Ltd. (Shenzhen, China) for analyzing the raw reads from sequencing, http://cloud.magigene.com) to examine the acquired raw reads. Specifically, a sliding window (average quality score = 20, window size = 4) was applied to screen the obtained raw reads using fastp software (V0.14.1, https://github.com/OpenGene/fastp) (Chen et al. 2018). We then utilized cutadapt software (V1.14, https://github.com/marcelm/cutadapt/) to remove the primers from the paired‐end reads. Afterwards, the acquired paired‐end clean reads were merged using usearch (V10, http://www.drive5.com/usearch/). All of the quality‐filtered sequences were clustered into operational taxonomic units (OTUs) using UPARSE (V7.1, http://drive5.com/uparse) with 97% nucleotide identity (Edgar 2013). Representative sequences were taxonomically classified using the SILVA V132 (http://www.arb‐silva.de) database with a confidence cutoff of 80% (Quast et al. 2013). Finally, PICRUSt2 was applied to predict the potential function of bacteria according to OTU taxonomic information (Douglas et al. 2020). Moreover, all the OTUs were classified based on their relative abundances to determine rare taxa. We defined relative abundance thresholds as 0.01% for rare taxa by referring to previous literature (Liu et al. 2015).

### 2.4 Water Environment, Nutrient, and Spectral Analyses

The pH, conductivity, and dissolved oxygen (DO) were measured in the field using a multi‐parameter water quality analyzer (YSI, Yellow Springs, USA). Ammonium (NH_4_^+^‐N) and nitrate (NO_3_^−^‐N) contents were examined using a SEAL AA3 continuous flow analyzer (SEAL Analytical Ltd., Southampton, UK) after conducting a standard curve with NH_4_^+^‐N and NO_3_^−^‐N mother liquor (Hu et al. 2023). Total dissolved phosphorus (TDP) in the water was measured using an ICAP 6300 inductively coupled plasma spectrometer (Thermo Fisher Scientific Inc., Waltham, USA) after digestion (120°C) with K_2_S_2_O_8_ (Li et al. 2023). Both the dissolved organic carbon (DOC) and total dissolved nitrogen (TDN) were determined using a multi‐NC‐3100 analyzer (Analytik Jena Ltd., Jena, Germany) after establishing a standard curve with DOC and TDN mother liquor (Hu et al. 2023).

The spectral characteristics of dissolved organic matter were measured using a UV–visible spectrophotometer (Lambda35, Perkin Elmer Inc., Waltham, USA) and a fluorescence spectrophotometer (F‐7000, Hitachi Ltd., Tokyo, Japan). Specifically, the optical parameters including a_300_, SUVA_254_, and S_275‐295_ were determined based on the absorbance scanned between 200 and 600 nm wavelength. a_300_ was obtained by multiplying the absorbance (A) at 300 nm by the Napier absorption coefficient (2.303) and dividing by the path length (0.01 m). This parameter was used to denote the content of chromophoric dissolved organic matter (Cory et al. 2013). SUVA_254_, a proxy representing the aromaticity of dissolved organic matter, was calculated by dividing the absorption coefficient at 254 nm by the DOC concentration (Weishaar et al. 2003). Spectral slope (S_275–295_), a typical index indicating the molecular weight of DOC, was determined using the following equation (Helms et al. 2008).

(1) $$\alpha_{\lambda }=\alpha_{\text{λref}}\;e^{-S\left(\lambda -\text{λref}\right)}$$

where α (m^−1^) denotes the Naperian absorption coefficient at λ wavelength (nm), and λref represents the reference wavelength (nm).

Excitation‐Emission matrix (EEM) was determined with an F‐7000 fluorometer (Hitachi Ltd., Tokyo, Japan). Then, both the biological index (BIX) and humification index (HIX) were calculated based on EEM. Specifically, BIX is usually used to indicate newly generated or locally sourced DOC, obtained by the emission fluorescence intensity at 380 nm divided by that at 430 nm under the 310 nm excitation wavelength (Li et al. 2023). HIX is commonly applied to denote the degradation degree of DOC, obtained by the ratio of the intensity of fluorescence emission at 435–480 nm to the sum of the intensities of fluorescence emission at 300–345 and 435–480 nm under the 254 nm excitation wavelength (Fellman et al. 2010).

Based on the above analyses, we could obtain the parameters including environments, nutrients, and substrates from the streams, tributaries, and main stems of the Yangtze River Source Region (Table S2). Besides these three types of factors, we also acquired data including land use, basin geometry, and human activity from the GLC_FCS30 (Zhang et al. 2021) and the Resource and Environmental Science Data Platform (https://www.resdc.cn/DOI/doiList.aspx), respectively (Table S3).

### 2.5 Statistical Analyses

All data were examined for normality and homoscedasticity before analysis and transformed when necessary. Firstly, one‐way analyses of variance (ANOVAs) with least significant difference (LSD) multiple comparisons were conducted to examine the differences in bacterial community composition, alpha diversity, beta diversity, and functional potentials along the streams, tributaries, and main stems of the Yangtze River Source Region. Nonmetric multidimensional scaling analysis (NMDS) and permutational multivariate ANOVA (PERMANOVA) were used to analyze the differences in bacterial community structure between the streams, tributaries, and main stems of the rivers. Secondly, the Mantel test, variation partitioning, and canonical correspondence analyses were performed to reveal the effects of explained variables on bacterial communities in the fluvial networks. Finally, we used the Stegen framework to evaluate the contribution of several ecological processes in regulating bacterial community assembly based on the β‐nearest taxon index (βNTI) and Bray‐Curtis‐based Raup‐Crick (RC_Bray_) (Stegen et al. 2013). Bacterial community assembly could be divided into five processes, including heterogeneous selection (βNTI values > 2), homogeneous selection (βNTI values < −2), dispersal limitation (|βNTI| values < 2, RC_Bray_ > 0.95), homogenizing dispersal (|βNTI| values < 2, RC_Bray_ < −0.95), and drift (|βNTI| values < 2, |RC_Bray_| < 0.95). The former two processes represent deterministic processes, while the other three processes represent stochastic processes. All the above statistical analyses were conducted in the software R version 4.0.4. Network analysis was conducted as follows: pairwise Spearman's correlations between bacterial communities (OTU levels) were analyzed, and representative OTUs with significant correlation (Spearman's coefficient > 0.8 and *p* < 0.01) were selected and finally visualized using Gephi software (V0.10.1 https://gephi.org/).

## 3 Results

### 3.1 Changes in the Riverine Bacterial Community Composition and Structure

**FIGURE 2:**
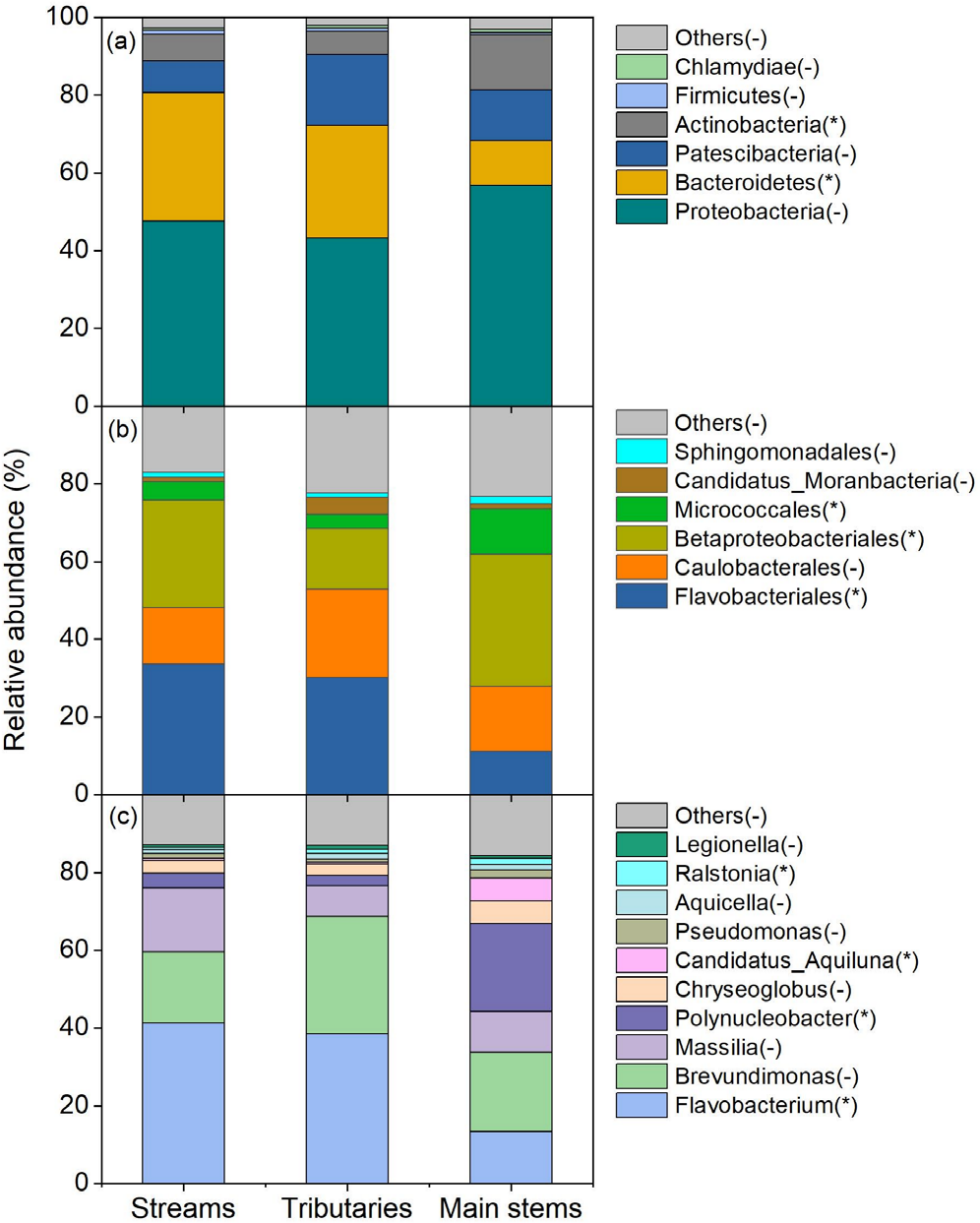
Composition of bacterial communities in streams, tributaries, and main stems of the Yangtze River Source Region. Panels (a–c) represent the relative abundances of bacterial communities at the phylum, order, and genus levels, respectively. The asterisk (*) in the parentheses of the legend indicates significant differences existing in the bacterial community composition between these three levels of stream orders (LSD test, *p* < 0.05), while the horizontal line (−) denotes no significant differences among them (LSD test, *p* > 0.05).

Bacterial community composition in the Yangtze River Source Region was mainly composed of *Proteobacteria*, *Bacteroidetes*, *Patescibacteria*, and *Actinobacteria* (Figure 2a). Among them, the phylum *Proteobacteria* was dominant in the bacterial communities, accounting for more than 50%. The relative abundances of *Bacteroidetes*, *Patescibacteria*, and *Actinobacteria* were 24%, 13%, and 9%, respectively. By comparison, other types of bacteria accounted for less than 5%. In these fluvial networks, the bacterial composition in streams was similar to that in tributaries. By comparison, the relative abundance of *Bacteroidetes* significantly decreased, while that of *Actinobacteria* significantly increased in main stems (based on one‐way ANOVAs with LSD test; all *p* < 0.05). At the order level, the relative abundance of *Flavobacteriales* significantly declined while those in *Betaproteobacteriales* and *Micrococcales* significantly enhanced along the stream orders (based on one‐way ANOVAs with LSD test; all *p* < 0.05; Figure 2b). Similarly, at the genus level, the relative abundance of *Flavobacterium* significantly decreased, while those in *Polynucleobacter*, *Candidatus_Aquiluna*, and *Ralstonia* significantly increased along the stream orders (based on one‐way ANOVAs with LSD test; all *p* < 0.05; Figure 2c), indicating that significant differences existed in the bacterial community composition in the stream orders (stream‐tributary‐main stem), whether at the phylum, order, or genus levels.

**FIGURE 3:**
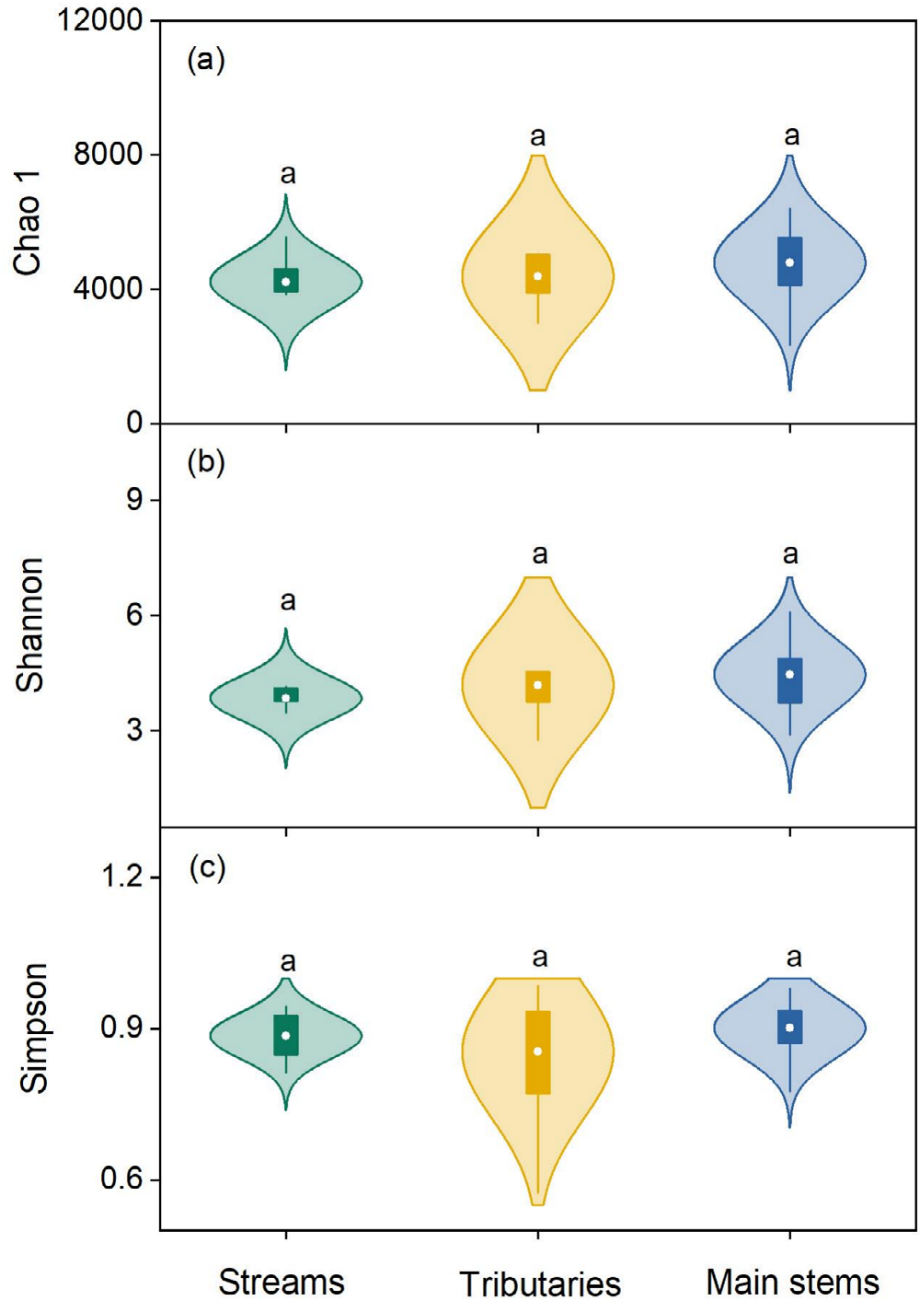
Comparisons of bacterial alpha diversity at the different stream orders of the Yangtze River Source Region. (a) Bacterial Chao1 index. (b) Bacterial Shannon diversity. (c) Bacterial Simpson's diversity. The same lowercase letters represent no significant difference (LSD test, *p* > 0.05) in bacterial alpha diversity among the three stream orders. The whiskers within violin plots denote the 5th and 95th percentiles, and the box ends illustrate the 25th and 75th percentiles (interquartile range). The circles in the boxes indicate the mean values of the data (*n* = 9), respectively.

**FIGURE 4:**
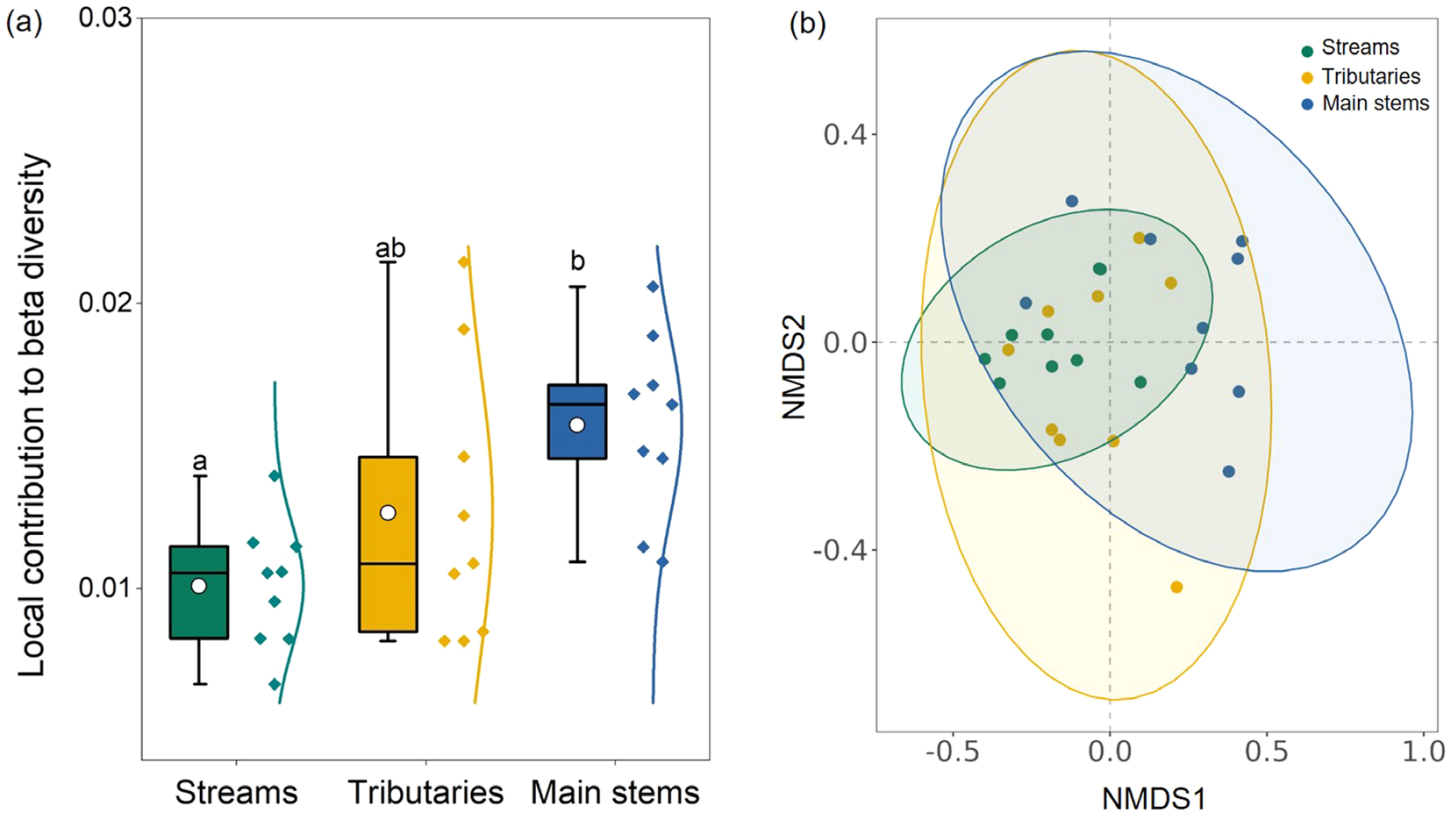
Changes in local contribution to beta diversity of bacteria and community structure along the streams, tributaries, and main stems of the Yangtze River Source Region. (a) Bacterial beta diversity index. Different lowercase letters indicate significant differences in bacterial beta diversity among the different stream orders (LSD test, *p* < 0.05). The whiskers within violin plots denote the 5th and 95th percentiles, and the box ends illustrate the 25th and 75th percentiles. The lines and circles in the boxes indicate the median and mean values of the data (*n* = 9), respectively. The lines on the right side of the box represent the normal distribution curve based on the data of bacterial beta diversity. (b) Nonmetric multidimensional scaling (NMDS) analysis of bacterial community structure. Ellipses denote 95% confidence levels for bacterial communities in each stream order.

**FIGURE 5:**
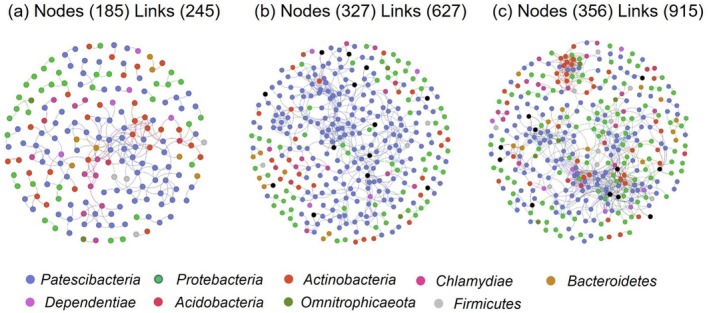
Network analysis for bacterial communities in the streams (a), tributaries (b), and main stems (c) of the Yangtze River Source Region. Different color circles represent different phyla of bacteria.

The bacterial alpha diversity, including Chao1, Shannon, and Simpson indexes, showed no significant differences among the streams, tributaries, and main stems of the Yangtze River (LSD test; all *p* > 0.05; Figure 3). Different from bacterial alpha diversity, the beta diversity gradually increased along the fluvial networks (Figure 4a). Furthermore, the bacterial community structure was also different between these three stream orders (Figure 4b and Table 1). Specifically, the structure of bacterial communities in the main stems of rivers was significantly different from that in streams and tributaries. Network analysis further showed that the number of nodes and links between bacterial communities largely increased along the stream orders, suggesting an enhanced complexity of bacterial communities (Figure 5). Furthermore, we also analyzed the characteristics of rare bacteria along the streams, tributaries, and main stems of the Yangtze River. The results illustrated that there were no significant differences in the abundance and diversity of rare taxa between these three levels of stream orders (Figure S1), indicating that rare taxa exhibit minimal variation along the stream orders. However, the predicted functional potential of bacteria for carbon and nitrogen cycling gradually decreased from streams to main stems of the rivers, especially for the nitrogen and methane metabolisms (Figure S2).

**TABLE 1:** PERMANOVA for bacterial community structure along the streams, tributaries, and main stems of the Yangtze River Source Region.

	*F*	*p*
Streams vs. tributaries	1.726	0.071
Streams vs. main stems	5.772	**0.002**
Tributaries vs. main stems	3.401	**0.003**

*Note: F* value is calculated based on PERMANOVA, representing the difference in variances between two groups of samples. *p* Value denotes the statistical significance. Significant differences (*p* < 0.05) are denoted in bold.

### 3.2 Dominant Driver Influencing the Riverine Bacterial Communities

**FIGURE 6:**
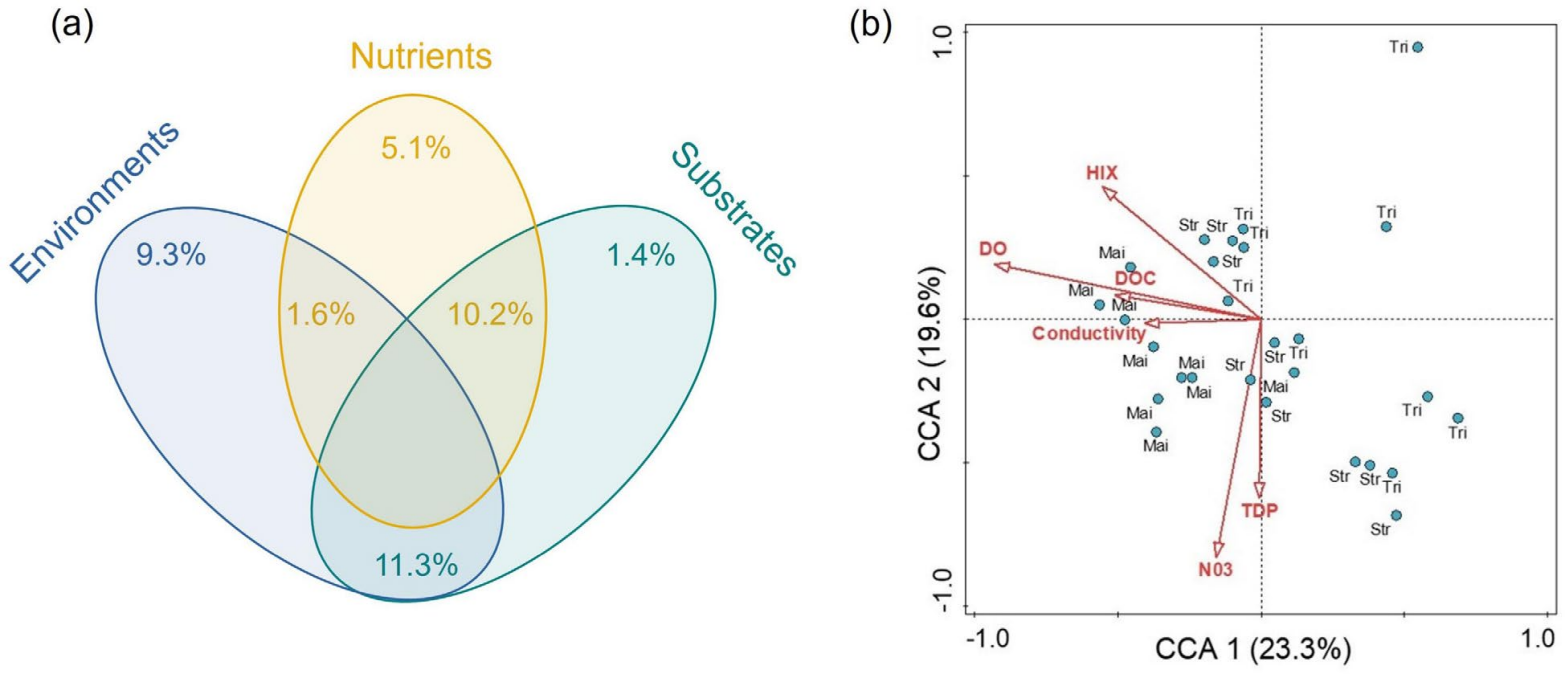
Variation partitioning (a) and canonical correspondence analyses (b) for the effects of environments, nutrients, and substrates on the bacterial communities in the Yangtze River Source Region. DO, dissolved oxygen; DOC, dissolved organic carbon; HIX, humification index; Mai, main stem; NO_3_, nitrate; Str, stream; TDP, total dissolved phosphorus; Tri, tributary.

The Mantel test denoted that environments, nutrients, and substrates exerted significant effects on the bacterial communities in the Yangtze River Source Region (Table 2), while land use, basin geometry, and human activity intensity had less effects on bacteria (Table S4). Specifically, with respect to environments, both DO and conductivity significantly affected the bacteria (all *p* < 0.05), while pH had less influence on them (*p* > 0.05). Regarding the nutrient variables, the contents of NO_3_^−^‐N and TDP exhibited significant effects on bacterial communities (all *p* < 0.05), while TDN and NH_4_^+^‐N showed limited effects on them (all *p* > 0.05). For the substrates, the concentration of DOC and its degradation degree (HIX) significantly influenced the bacteria (all *p* < 0.05). Although these three above‐mentioned factors could affect the bacterial communities, variation partitioning analysis illustrated that environmental variables were the dominant factor regulating the riverine bacteria (Figure 6a). The canonical correspondence analysis further confirmed this observation, revealing that compared to nutrients and substrates, water environments (i.e., DO) primarily influenced the bacterial communities along the stream orders (Figure 6b).

**TABLE 2:** Relationships of bacterial communities with environments, nutrients, and substrates in the Yangtze River Source Region revealed by the Mantel test.

Type	Parameters	*r*	*p*
Environments	DO	0.189	**0.017**
	pH	−0.136	0.937
	Conductivity	0.158	**0.048**
Nutrients	TDN	0.016	0.370
	NH_4_^+^‐N	0.007	0.417
	NO_3_^−^‐N	0.128	**0.043**
	TDP	0.149	**0.046**
Substrates	DOC	0.133	**0.045**
	S_275‐295_	0.040	0.313
	SUVA_254_	0.031	0.323
	a_300_	0.061	0.246
	BIX	−0.005	0.491
	HIX	0.237	**0.012**

*Note: r* represents the correlation coefficient. *p* Value denotes the statistical significance. Significant differences (*p* < 0.05) are denoted in bold.

Abbreviations: a_300_ = Naperian absorption coefficient at 300 nm (m^−1^), BIX = biological index, DO = dissolved oxygen (mg L^−1^), DOC = dissolved organic carbon (mg L^−1^), HIX = humification index; NH_4_^+^‐*N* = ammonium (mg L^−1^), NO_3_^−^‐*N* = nitrate (mg L^−1^), S_275‐295_ = spectral slope (×10^−3^ nm^−1^), SUVA_254_ = specific UV absorbance at 254 nm (L mg C^−1^ m^−1^), TDN = total dissolved nitrogen (mg L^−1^), TDP = total dissolved phosphorus (μg L^−1^).

### 3.3 Deterministic Processes Shaping the Bacterial Communities

**FIGURE 7:**
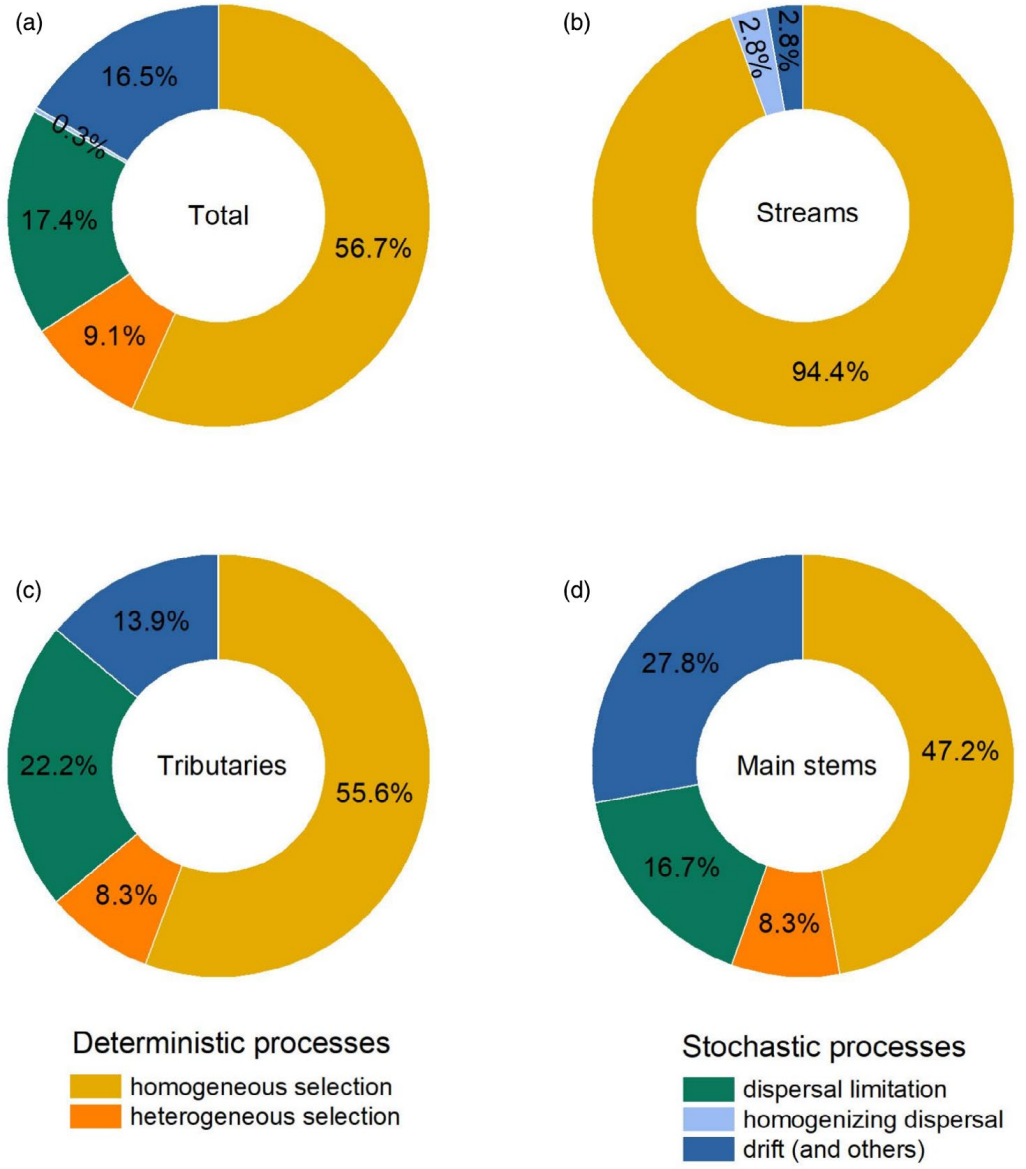
Relative contribution of each process controlling the bacterial community assembly within different stream orders in the Yangtze River Source Region based on null model analysis: Total rivers (a), streams (b), tributaries (c), and main stems (d). Different colors represent stochastic and deterministic processes driving the bacterial community assembly.

Deterministic processes exerted a dominant role in regulating bacterial communities of the Yangtze River Source Region (Figure 7a). The relative contributions of homogeneous selection and heterogeneous selection were 56.7% and 9.1%, while those in dispersal limitation, drift, and homogenizing dispersal were 17.4%, 16.5%, and 0.3%, respectively. Specifically, the bacterial community assembly was also primarily driven by homogeneous selection, whether in the streams, tributaries, or main stems of the river (Figure 7b–d). However, the relative importance of homogeneous selection decreased from 94.4% to 47.2% along the stream orders. By contrast, stochastic processes, such as drift, had an unneglectable contribution in structuring the bacterial communities, which gradually increased from 2.8% to 27.8% along the stream orders.

## 4 Discussion

### 4.1 Differences Existed in the Bacterial Community Composition and Beta Diversity

Our results illustrated that the bacteria in the Yangtze River Source Region were mainly composed of *Proteobacteria*, *Bacteroidetes*, *Patescibacteria*, and *Actinobacteria* (Figure 2). This observation was supported by previous studies conducted in other rivers (Gao et al. 2017; Liu et al. 2018), indicating that these above‐mentioned microorganisms belong to the dominant phylum in freshwater. These four types of bacteria could exert crucial roles in DOC decomposition and nutrient cycling, which are major participants in biogeochemical cycles (Gao et al. 2017; Zhang et al. 2019). In addition, the bacterial community composition in the main stems was different from that in the streams and tributaries, with a significant decrease in the relative abundance of *Bacteroidetes* but an increase in *Actinobacteria*, supporting our first hypothesis. This difference may be ascribed to environmental changes in these three stream orders of the Yangtze River. Our results demonstrated that DO significantly increased from the headwater streams to the main stems of rivers (Figure S3). Given that *Bacteroidetes* are mostly obligate or facultative anaerobic bacteria (Lapébie et al. 2019), they would be reduced due to unfavorable environmental changes such as increased oxygen. In contrast, *Actinobacteria* mostly belong to aerobic bacteria, which are more suitable for their growth in a higher DO condition (Garcia et al. 2013). Therefore, the increased DO along the three levels of stream orders may result in shifts in bacterial community composition. Such an explanation was further supported by the variation partitioning and canonical correspondence analyses (Figure 6), revealing that DO was the dominant factor regulating the changes of bacterial communities along the streams, tributaries, and main stems of the Yangtze River.

Our results also demonstrated that no significant differences existed in the bacterial alpha diversity along the stream orders (Figure 3), while the beta diversity significantly increased (Figure 4), which also supported the first hypothesis in this study. Although the bacterial richness and evenness exhibited no obvious changes in these fluvial networks, their community structure shifted along these three stream orders, indicating that bacterial community structure is more sensitive to environmental changes in the Yangtze River. Generally, the beta diversity could reflect the dissimilarity of species composition between different habitat communities along environmental gradients (Zhong et al. 2021). In other words, the bacterial community structure becomes more complex along the stream orders (Anderson et al. 2011; Socolar et al. 2016). This inference was also verified by the network analysis conducted in this study (Figure 5), revealing that the links between bacterial communities gradually increased along the streams, tributaries, and main stems of rivers. This finding was also supported by a recent study. Based on 1235 global observations, Zhou et al. (2020) found that some global change factors (such as increased atmospheric temperature and CO_2_ concentration) exerted less effects on microbial alpha diversity but significantly altered microbial beta diversity, suggesting that the latter is more sensitive to environmental changes compared with the former.

### 4.2 Environments Dominantly Mediated the Bacterial Communities

Our results showed that environments, rather than substrates and nutrients, were the primary factor resulting in the differences in bacterial communities along the stream orders, supporting our second hypothesis. It has been reported that both DO and conductivity (the relationship between these two parameters see Note S1) are important factors affecting microbial communities (Liu et al. 2018; Wu et al. 2022; Zhao et al. 2023). Oxygen can serve as an electron acceptor in bacterial degradation of organic matters, participating in the energy metabolism and subsequent microbial growth (Oosterkamp et al. 2013). Similarly, higher conductivity means more electron transporters in rivers, which is also more conducive for microorganisms engaging in material cycles and energy metabolism (Kumar et al. 2019). Therefore, the environments including DO and conductivity primarily regulated the composition and structure of bacterial communities. However, this study illustrated that pH exhibited little effects on the riverine bacteria, which was different from previous findings. It has been reported that pH plays a vital role in proton pumps and protein stability that bind to cell membranes (Booth 1985). When pH exceeds a certain range (ecological niche), it can cause physiological stress on microorganisms, and result in a rapid decrease in some microbial groups and ultimately alter their community composition and structure (Zhou et al. 2020). However, in our case, there were no significant changes in pH along the three stream orders (Figure S3), consequently leading to limited effects of pH on bacterial communities. Moreover, bacterial cell membranes have a certain buffering capacity, which can resist the changes of pH on microbial metabolism to a certain extent (Rius and Lorén 1998; Krulwich et al. 2011). Especially in ecosystems with little variation in pH, it may exhibit a weaker effect on the bacterial communities.

Besides the environments, substrates and nutrients may also impact the bacteria. Numerous previous studies showed that the microbial community composition and metabolic strategies (such as *r*‐ and *K*‐strategists) were closely related to substrates (Fontaine et al. 2003), illustrating that both the substrate quantity and quality can influence the microbial community and diversity (Zhou et al. 2011; Billings and Ballantyne 2013). Accompanying the decreased quantity (i.e., DOC) and quality (i.e., HIX) of substrates, microbial communities would shift to degrade the recalcitrant substances, which adapt to resource variability and maintain energy acquisition (Fontaine et al. 2003; Feng et al. 2017). In addition, the role of nutrients (including inorganic nitrogen and phosphorus) in microorganisms cannot be ignored. Microorganisms are generally limited by inorganic nitrogen and phosphorus, especially in high‐altitude areas (Myrstener et al. 2018; Li et al. 2023). Given that low temperature limits the cycling of nitrogen and phosphorus, their content in water bodies is generally low (Qu et al. 2018; Zhang et al. 2019). Thus, the low concentration of inorganic nitrogen and phosphorus could in turn affect the microbial normal metabolism and finally alter microbial community composition and diversity (Smith and Prairie 2004; Li et al. 2023). However, this study found that, compared to the environments, both substrates and nutrients had weaker effects on bacterial communities, indicating that bacterial communities in the Yangtze River Source Region are more sensitive to environmental changes. Substrates and nutrients may affect bacteria through the joint effects with environments (Liu et al. 2021), which was supported by the large overlap between these three types of factors (Figure 6a).

The observation that environments instead of substrates and nutrients mainly regulated bacterial communities could be explained by niche theory. This theory emphasizes that the changes in community composition are the result of combined effects of environmental filtering and competitive exclusion (Hirzel and Lay 2008). Although nutrients and substrates are the basis for microbial growth, environments (i.e., oxygen, temperature and pH) dominantly shape the microbial communities (Fierer and Jackson 2006; Wu et al. 2022). Specifically, environmental factors directly define the survival boundary of bacterial communities. For instance, aerobic bacteria cannot survive in strictly anaerobic environments, regardless of substrate and nutrient availability (Conrad 2007). Environmental conditions dominate the threshold for microbial survival, and breaking the threshold may result in microbial function loss or even death, while substrate and nutrient availability are only further optimized within their tolerance range (Fierer and Jackson 2006; Zhao et al. 2023). Moreover, environmental factors may affect the niche of microbial community resource utilization. Even if both the substrates and nutrients are the same, environmental factors can reshape the community structure by changing the microbial metabolic efficiency and competitive strategies. For example, at low temperatures, the enzyme catalytic rate of mesophilic bacteria significantly decreases, while psychrophilic bacteria maintain their substrate conversion advantage through flexible protein structures and cold‐shock proteins (D'Amico et al. 2006). Taken together, environmental factors, as the “primary filter”, determine the survival and metabolic strategies of microorganisms (Fierer and Jackson 2006; Hirzel and Lay 2008; Liu et al. 2024). Both nutrients and substrates might act as “secondary regulators”, refining interspecific competition and coexistence patterns only to the extent permitted by the environments. Therefore, compared with nutrients and substrates, environments played greater roles in shaping the bacterial communities.

### 4.3 Deterministic Processes Primarily Regulated the Bacterial Community Assembly

Our results indicated the prominent role of deterministic processes in regulating the bacterial communities (Figure 7), which supported our third hypothesis. Such a phenomenon may be attributed to the harsh environment of the Yangtze River Source Region. Both the Mantel test and canonical correspondence analysis further confirmed that the environment was the primary driver in shaping the bacterial communities (Table 2 and Figure 6). Specifically, our study area is located on the Tibetan Plateau; environmental stresses induced by low temperature and oxygen can exclude the species that are not adapted to harsh environments and then lead to stronger niche‐selection effects (Dini‐Andreote et al. 2014; Siqueira et al. 2020). Therefore, deterministic processes, rather than stochastic processes, dominated the bacterial community assembly. However, the contribution of stochastic processes, especially the drift in regulating the bacterial community, gradually increased along the stream orders. The increased stochastic processes might be due to environmental disturbances. Compared to streams and tributaries, the environmental changes in the main stems of rivers are more drastic (Figure S3). These considerable environmental disturbances induce the decimation of local microbial populations (Darcy et al. 2011), which may strengthen the ecological drift and ultimately increase the stochastic processes (Kang et al. 2021). Consequently, heterogeneity resulting from stream order may act as an environmental filter, which finally results in compositional variations among the bacterial communities.

Consistent with this finding, previous studies conducted in the Yangtze River watershed also illustrated that deterministic processes predominantly governed the bacterial community assembly (Wu et al. 2022; Liu et al. 2024). However, some research involving the bacterial community assembly in plain river systems demonstrated that stochastic processes rather than deterministic processes primarily shaped the communities (Chen et al. 2019; Ma et al. 2022; Zhu et al. 2024). This discrepancy could be ascribed to the different altitudes of sampling areas. It has been reported that the variations of bacterial communities are closely associated with elevation (Liu et al. 2024). Due to the harsh environment caused by high altitude, deterministic processes mainly regulated the bacterial community assembly. This deduction was also supported by the research conducted in other high‐altitude areas, revealing deterministic processes dominating the bacterial community structure in rivers on the Mongolia Plateau (Tang et al. 2021). By contrast, stochastic processes exerted more contribution in driving bacterial community assembly in low‐altitude rivers. Such a phenomenon could be attributed to the following two aspects. Firstly, human activities in low‐altitude regions are stronger than those in high altitude and thus result in urban sewage and agricultural pollutants entering rivers through surface runoff (Liu et al. 2024). These environmental disturbances could trigger massive deaths of local microbial populations (Darcy et al. 2011) and subsequent ecological drift (Kang et al. 2021), increasing the role of stochastic processes in driving the structure of bacterial communities. Secondly, low‐altitude rivers contain numerous reservoirs and water diversion projects and thus form hydrologically isolated flow paths (Zhu et al. 2024), which may contribute to dispersal limitation and ultimately strengthen the contribution of stochastic processes. Overall, given the differences in environments induced by elevation variations, the community assembly in low‐altitude rivers was controlled by the stochastic processes, while that in high‐altitude rivers was primarily regulated by the deterministic processes.

In summary, the dominant bacteria in the upper reaches of the Yangtze River mainly consisted of *Proteobacteria*, *Bacteroidetes*, *Patescibacteria*, and *Actinobacteria*, accounting for over 95% of the bacterial community composition. Despite no significant changes in microbial alpha diversity, their beta diversity significantly increased along the three stream orders (stream‐tributary‐main stem), enhancing the microbial complexity of bacterial communities. Furthermore, our results illustrated that compared to nutrients and substrates, water environments were the dominant factor shaping the riverine bacteria. Deterministic processes played a dominant role in regulating bacterial community assembly, regardless of whether in streams, tributaries, or main stems of rivers. In addition, we found that the ability of microbial carbon and nitrogen metabolism gradually decreased along these three stream orders (Figure S2). Such a phenomenon indicated that the bacteria from the tributaries could exert stronger element cycles and ecological processes than those in the main stems of rivers, despite the lower microbial abundance and beta diversity in the former. If the microorganisms from streams or tributaries are disregarded, the carbon and nitrogen cycles performed by microorganisms could be seriously underestimated, which in turn affects the assessment of river ecosystem functions. Taken together, this study highlighted that the ecological processes mediated by microbes from streams and tributaries should be integrated into the Earth system models, which would be more beneficial to accurately predict the response of aquatic ecosystems to climate change.

While this study explored riverine bacterial communities along the stream orders of the Yangtze River, some limitations still exist and need to be addressed in future studies. First, only one time of water samples collected for one season was applied for examining bacterial communities in this study, which can only reveal the bacterial dynamics in summer. The characteristics of bacterial communities across the seasonal scale should be further studied via multiple times of sampling in other seasons. Second, due to the limitations of field sampling, we did not collect river sediments and soils around the sampling zone, and thus we could not analyze the sources of riverine plankton bacteria. In future research, microbial data from river sediments and soils around the sampling area should also be combined to study the sources of bacterial communities.

## Author Contributions

**Futing Liu:** conceptualization (lead), data curation (lead), formal analysis (lead), funding acquisition (lead), methodology (lead), supervision (lead), writing – original draft (lead), writing – review and editing (lead). **Luyao Kang:** formal analysis (equal), methodology (equal), writing – review and editing (equal). **Lele Lin:** formal analysis (equal), writing – review and editing (equal). **Sisi Yu:** data curation (equal), funding acquisition (equal), writing – review and editing (equal).

## Conflicts of Interest

The authors declare no conflicts of interest.

## Supporting information


Data S1.

## Data Availability

All data are uploaded in figshare at https://doi.org/10.6084/m9.figshare.27173469.v1.
