# Supplementary material for: Microbial Community Characteristics and Underlying Drivers Along the Streams, Tributaries, and Main Stems of the Yangtze River Source Region
Source: Ecol Evol. 2025 Apr 15;15(4):e71290. doi: 10.1002/ece3.71290 (PMC11997365; doi:10.1002/ece3.71290)
Supplement: Supplementary file 1 — Data S1. [file ECE3-15-e71290-s001.docx]

**Supplementary Information**

**Microbial Community Characteristics and Underlying Drivers Along the Streams, Tributaries, and Main Stems of the Yangtze River** **Source Region**

Futing Liu^1,2*^, Luyao Kang^2^, Lele Lin^1^, Sisi Yu^1^

^1^Key Laboratory of Forest Ecology and Environment of National Forestry and Grassland Administration, Ecology and Nature Conservation Institute, Chinese Academy of Forestry, Beijing, China

^2^State Key Laboratory of Vegetation and Environmental Change, Institute of Botany, Chinese Academy of Sciences, Beijing, China

***Corresponding author:** Dr. Futing Liu, E-mail: [liufuting@caf.ac.cn](mailto:liufuting@caf.ac.cn)

**Note S1. Relationship between dissolved oxygen and conductivity**

Dissolved oxygen (DO) and conductivity may be indirectly correlated through mediating variables such as temperature, salinity or biochemical processes (*i.e.* microbial decomposition). Specifically, DO refers to the amount of oxygen dissolved in water. It is a critical indicator reflecting purification capacity of water bodies and ecosystem health (aerobic organism survival) (Li et al. 2023). Conductivity could be used to reflect the content of dissolved salts, minerals, and heavy metals (Jiménez et al. 2009). If the river is affected by sewage discharge, the decomposition of organic matter (such as eutrophication) will consume oxygen (DO reduction), while releasing ions (such as NH_4_^+^ and NO_3_^-^), leading to an increase in conductivity. However, previous studies illustrated that in freshwater ecosystems with no pollution, DO is affected by temperature, oxygen partial pressure and biological activity, while conductivity is mainly determined by carbonate and calcium magnesium ions (Jiménez et al. 2009; Li et al. 2023). There is no clear direct correlation between DO and conductivity. These two parameters may exhibit positive (Li et al. 2023; Liang et al. 2024), negative (Liu et al. 2020), or no significant correlations (Irvine et al. 2011). For instance, a recent research reported that DO has a significant positive correlation with conductivity. Another research also revealed that the linear correlation coefficient between DO and conductivity was 0.608 (Liang et al. 2024). These two studies further support our findings that DO was positively related to conductivity. Therefore, it was reasonable that both DO and conductivity were higher in the main stems of the Yangtze River compared to those in streams and tributaries.

**Table S1** Characteristics of sampling sites in the Yangtze River Source Region.

| Sampling  area | Stream order | Latitude  (^o^N) | Longitude  (^o^E) | Altitude  (m) | River width  (m) |
| --- | --- | --- | --- | --- | --- |
| Ulran Moron | Stream | 33.14 | 91.86 | 4838 | 35 |
|  | Stream | 33.31 | 91.90 | 4718 | 31 |
|  | Stream | 33.47 | 91.95 | 4628 | 36 |
|  | Tributary | 33.87 | 92.20 | 4529 | 70 |
|  | Tributary | 33.87 | 92.32 | 4525 | 78 |
|  | Tributary | 33.88 | 92.43 | 4484 | 89 |
|  | Main stem | 34.23 | 92.38 | 4499 | 104 |
|  | Main stem | 34.22 | 92.43 | 4492 | 110 |
|  | Main stem | 34.21 | 92.45 | 4489 | 122 |
| Qumarlêb | Stream | 34.56 | 95.96 | 4428 | 15 |
|  | Stream | 34.54 | 95.87 | 4366 | 18 |
|  | Stream | 34.55 | 95.68 | 4273 | 17 |
|  | Tributary | 34.55 | 95.51 | 4187 | 85 |
|  | Tributary | 34.56 | 95.44 | 4166 | 81 |
|  | Tributary | 34.53 | 95.37 | 4140 | 92 |
|  | Main stem | 34.12 | 95.70 | 4039 | 403 |
|  | Main stem | 34.08 | 95.75 | 4028 | 820 |
|  | Main stem | 34.04 | 95.82 | 4017 | 961 |
| Yushu | Stream | 32.67 | 97.23 | 4207 | 14 |
|  | Stream | 32.72 | 97.22 | 4078 | 12 |
|  | Stream | 32.78 | 97.20 | 3990 | 10 |
|  | Tributary | 33.01 | 97.02 | 3629 | 39 |
|  | Tributary | 33.01 | 97.12 | 3575 | 33 |
|  | Tributary | 32.99 | 97.21 | 3519 | 36 |
|  | Main stem | 32.98 | 97.24 | 3486 | 105 |
|  | Main stem | 32.93 | 97.27 | 3480 | 110 |
|  | Main stem | 32.91 | 97.34 | 3455 | 113 |

**Table S2** Environment, nutrient, and substrate parameters from streams, tributaries, and main stems of the Yangtze River Source Region.

| Sampling area | Stream order | DO | pH | Cond | NH_4_^+^-N | NO_3_^-^-N | TDP | DOC | S_275-295_ | SUVA_254_ | a_300_ | BIX | HIX |
| --- | --- | --- | --- | --- | --- | --- | --- | --- | --- | --- | --- | --- | --- |
| Ulran Moron | Stream | 4.01 | 8.27 | 399 | 0.067 | 0.266 | 2.44 | 1.09 | 13.51 | 3.08 | 7.22 | 0.89 | 0.73 |
|  | Stream | 4.55 | 8.29 | 418 | 0.064 | 0.268 | 2.72 | 1.09 | 15.37 | 2.96 | 5.30 | 0.80 | 0.71 |
|  | Stream | 4.5 | 8.34 | 391 | 0.071 | 0.263 | 3.97 | 1.01 | 12.57 | 4.47 | 5.99 | 0.84 | 0.73 |
|  | Tributary | 4.53 | 8.21 | 361 | 0.065 | 0.302 | 5.28 | 0.83 | 10.24 | 4.43 | 4.84 | 0.80 | 0.64 |
|  | Tributary | 4.26 | 8.22 | 406 | 0.065 | 0.301 | 3.38 | 0.89 | 11.16 | 4.35 | 3.45 | 0.80 | 0.73 |
|  | Tributary | 4.12 | 8.24 | 359 | 0.068 | 0.261 | 3.84 | 0.93 | 12.57 | 3.65 | 4.61 | 0.81 | 0.77 |
|  | Main stem | 5.18 | 8.57 | 976 | 0.083 | 0.306 | 7.00 | 1.22 | 18.05 | 3.60 | 4.84 | 0.82 | 0.81 |
|  | Main stem | 5.69 | 8.65 | 713 | 0.082 | 0.289 | 3.94 | 1.71 | 13.01 | 3.39 | 8.06 | 0.86 | 0.78 |
|  | Main stem | 5.98 | 8.6 | 645 | 0.089 | 0.31 | 6.09 | 1.63 | 12.18 | 2.76 | 6.22 | 0.87 | 0.76 |
| Qumarlêb | Stream | 3.51 | 9.03 | 509 | 0.067 | 0.564 | 3.63 | 1.26 | 17.24 | 2.54 | 3.45 | 0.86 | 0.70 |
|  | Stream | 3.39 | 8.86 | 413 | 0.065 | 0.482 | 1.94 | 1.45 | 13.72 | 2.21 | 3.92 | 0.84 | 0.71 |
|  | Stream | 3.6 | 8.66 | 501 | 0.066 | 0.505 | 4.47 | 1.16 | 18.39 | 2.92 | 3.92 | 0.85 | 0.72 |
|  | Tributary | 3.35 | 8.36 | 587 | 0.07 | 0.133 | 2.63 | 1.47 | 16.61 | 2.60 | 6.91 | 0.81 | 0.85 |
|  | Tributary | 3.26 | 8.43 | 475 | 0.066 | 0.286 | 2.44 | 0.91 | 10.91 | 3.91 | 8.98 | 0.83 | 0.60 |
|  | Tributary | 3.17 | 8.89 | 470 | 0.071 | 0.474 | 2.50 | 0.96 | 16.14 | 2.61 | 4.61 | 0.78 | 0.70 |
|  | Main stem | 4.56 | 8.3 | 671 | 0.077 | 0.454 | 4.25 | 1.95 | 14.86 | 3.03 | 5.76 | 0.80 | 0.76 |
|  | Main stem | 4.46 | 8.49 | 626 | 0.07 | 0.482 | 5.66 | 1.29 | 19.28 | 2.55 | 3.68 | 0.83 | 0.78 |
|  | Main stem | 4.68 | 8.36 | 432 | 0.071 | 0.433 | 4.16 | 1.46 | 18.15 | 2.12 | 3.45 | 0.85 | 0.80 |
| Yushu | Stream | 2.79 | 8.28 | 350 | 0.096 | 0.455 | 9.31 | 0.95 | 8.35 | 3.46 | 4.61 | 0.84 | 0.68 |
|  | Stream | 2.85 | 8.26 | 357 | 0.101 | 0.473 | 6.41 | 0.89 | 12.89 | 3.15 | 3.68 | 0.90 | 0.68 |
|  | Stream | 2.99 | 8.38 | 248 | 0.085 | 0.488 | 5.25 | 0.87 | 7.71 | 3.67 | 5.53 | 0.89 | 0.61 |
|  | Tributary | 2.92 | 8.35 | 471 | 0.067 | 0.342 | 4.28 | 0.97 | 3.85 | 3.92 | 5.53 | 0.78 | 0.50 |
|  | Tributary | 3.05 | 8.34 | 416 | 0.07 | 0.33 | 5.25 | 0.90 | 6.91 | 4.97 | 6.22 | 0.81 | 0.61 |
|  | Tributary | 3.01 | 8.28 | 407 | 0.07 | 0.271 | 11.72 | 0.92 | 13.60 | 3.61 | 3.45 | 0.78 | 0.60 |
|  | Main stem | 3.92 | 8.27 | 976 | 0.071 | 0.33 | 6.09 | 1.60 | 12.18 | 2.93 | 6.22 | 0.85 | 0.77 |
|  | Main stem | 4.35 | 8.29 | 962 | 0.069 | 0.46 | 3.59 | 1.49 | 15.85 | 3.36 | 7.14 | 0.89 | 0.79 |
|  | Main stem | 4.85 | 8.21 | 993 | 0.073 | 0.465 | 4.53 | 1.70 | 16.82 | 5.46 | 4.15 | 0.80 | 0.80 |

Abbreviations: DO = dissolved oxygen (mg L^-1^); Cond = Conductivity (μS cm^-1^); NH_4_^+^-N = ammonium (mg L^-1^); NO_3_^-^-N = nitrate (mg L^-1^); TDP = total dissolved phosphorus (μg L^-1^); DOC = dissolved organic carbon (mg L^-1^); S_275-295_ = spectral slope (×10^-3^ nm^-1^); SUVA_254_ = specific UV absorbance at 254 nm (L mg C^-1^ m^-1^); a_300_ = Naperian absorption coefficient at 300 nm (m^-1^); BIX = biological index; HIX = humification index.

**Table S3** Site information related to land use, basin geometry, human activity intensity.

| Sampling  area | Stream order | Land use | Slope  (^O^) | Population  (people/km^2^) | GDP  (¥10000/ km^2^) |
| --- | --- | --- | --- | --- | --- |
| Ulran Moron | Stream | Bare areas | 0.33 | 0 | 10 |
|  | Stream | Grassland | 6.02 | 4 | 60 |
|  | Stream | Grassland | 0.72 | 4 | 60 |
|  | Tributary | Grassland | 0.44 | 4 | 58 |
|  | Tributary | Bare areas | 0.31 | 4 | 58 |
|  | Tributary | Grassland | 0.76 | 4 | 58 |
|  | Main stem | Bare areas | 0.26 | 4 | 58 |
|  | Main stem | Bare areas | 0.23 | 0 | 4 |
|  | Main stem | Bare areas | 0.37 | 0 | 6 |
| Qumarlêb | Stream | Grassland | 0.67 | 1 | 2 |
|  | Stream | Grassland | 2.23 | 0 | 0 |
|  | Stream | Grassland | 5.66 | 0 | 1 |
|  | Tributary | Bare areas | 1.85 | 0 | 0 |
|  | Tributary | Bare areas | 4.16 | 0 | 0 |
|  | Tributary | Grassland | 3.23 | 0 | 0 |
|  | Main stem | Bare areas | 2.83 | 0 | 1 |
|  | Main stem | Grassland | 6.12 | 0 | 0 |
|  | Main stem | Grassland | 1.93 | 0 | 0 |
| Yushu | Stream | Grassland | 14.58 | 9 | 12 |
|  | Stream | Grassland | 14.58 | 9 | 12 |
|  | Stream | Grassland | 7.61 | 11 | 14 |
|  | Tributary | Grassland | 2.80 | 21 | 27 |
|  | Tributary | Grassland | 3.36 | 7 | 9 |
|  | Tributary | Grassland | 4.98 | 9 | 11 |
|  | Main stem | Grassland | 9.43 | 10 | 13 |
|  | Main stem | Grassland | 5.56 | 6 | 7 |
|  | Main stem | Grassland | 14.86 | 6 | 7 |

*Note:* The land use data were derived from the GLC_FCS30 (Zhang et al. 2021). Both the basin geometry and human activity intensity data were obtained from the Resource and Environmental Science Data Platform (https://www.resdc.cn/DOI/doiList.aspx).

**Table S4** Relationships of bacterial communities with land use, basin geometry, human activity intensity in the Yangtze River Source Region revealed by the Mantel test.

| Parameters | *r* | *p* |
| --- | --- | --- |
| Land use | 0.089 | 0.126 |
| Slope | 0.067 | 0.241 |
| Population | 0.017 | 0.401 |
| GDP | -0.079 | 0.819 |

*Note: r* represents the correlation coefficient. *p* Value denotes the statistical significance. Statistical differences were considered to be significant at the level of *P* < 0.05.

**
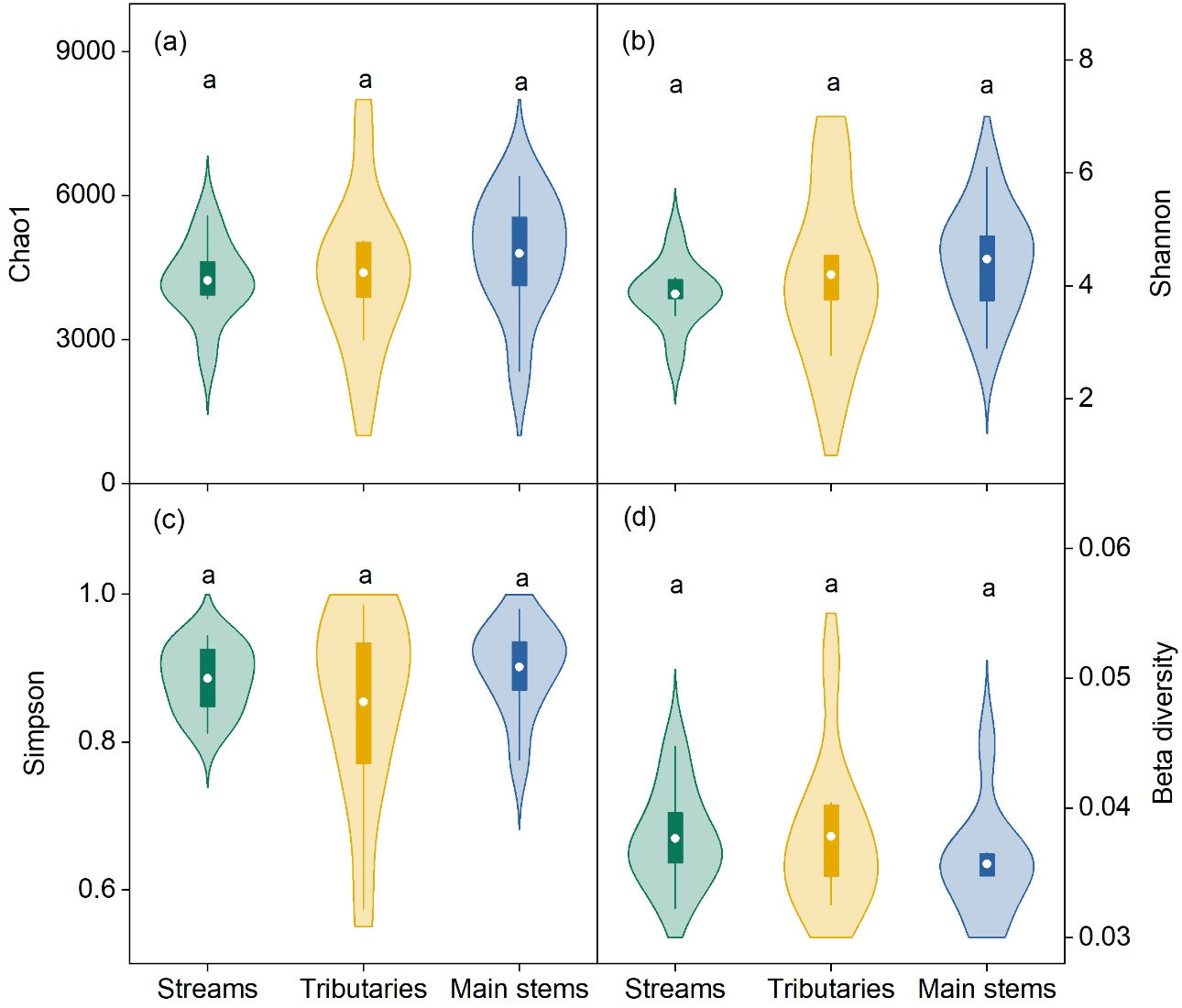
Fig. S1** Comparisons of rare bacterial alpha and beta diversity at the different stream orders of the Yangtze River Source Region. a, Rare bacterial Chao1 index. b, Rare bacterial Shannon diversity. c, Rare bacterial Simpson's diversity. d, Rare bacterial β diversity. The same lowercase letters represent no significant difference (LSD test, *P* > 0.05) in rare bacterial alpha and beta diversity among the three levels of stream orders. The whiskers within violin plots denote the 5th and 95th percentiles, and the box ends illustrate the 25th and 75th percentiles (interquartile range). The circles in the boxes indicate the mean values of the data (*n* = 9), respectively.

**
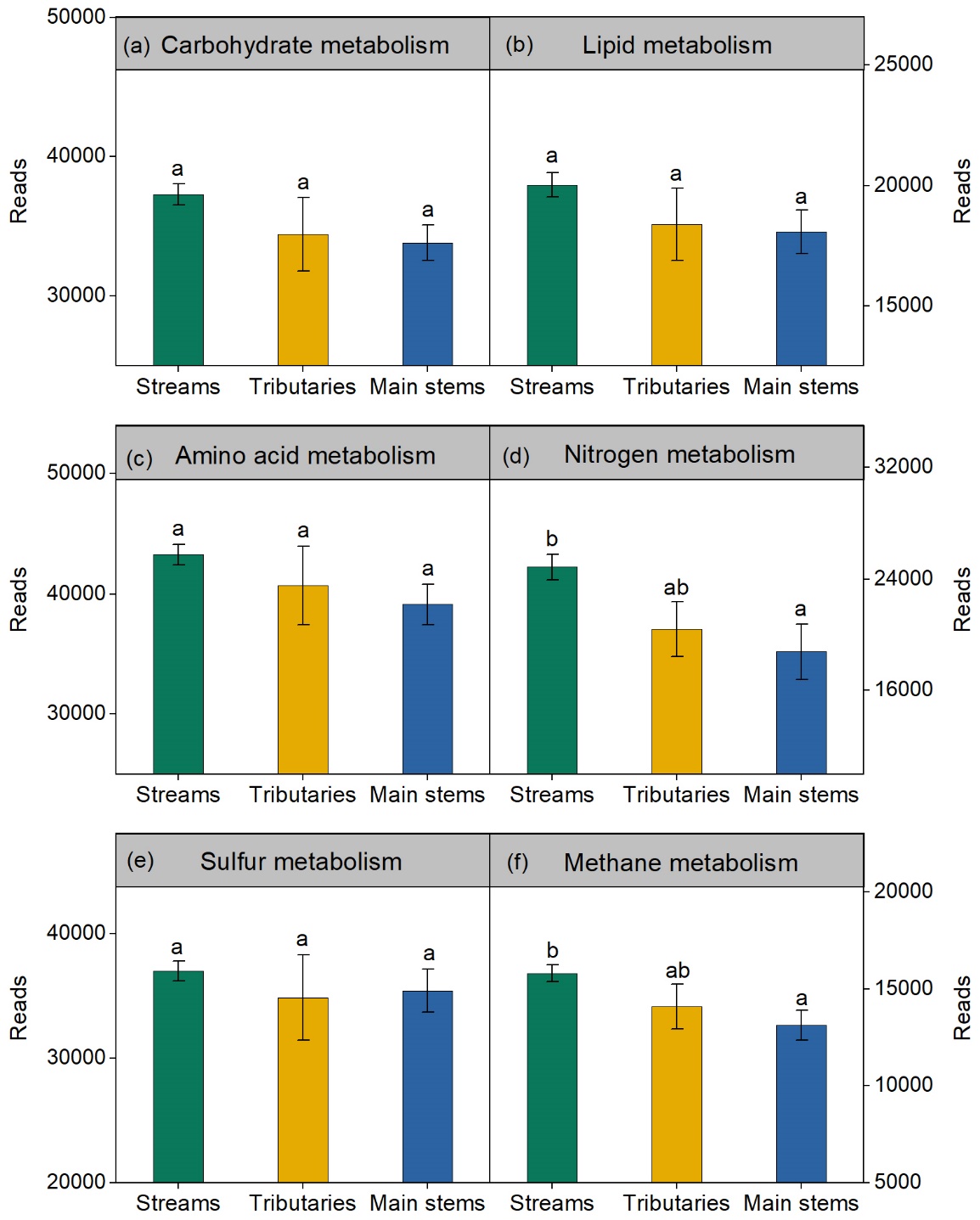
Fig. S2** Predicted functional potential of bacterial communities associated with carbohydrate (a), lipid (b), amino acid (c), nitrogen (d), sulfur (e), and methane (f) metabolisms in the Yangtze River Source Region. The bacterial functional potential was conducted by PICRUSt2 software, referring to the KEGG (Kyoto encyclopedia of genes and genomes) database. Different lowercase letters indicate significant differences in reads among the different stream orders (LSD test, *P* < 0.05).


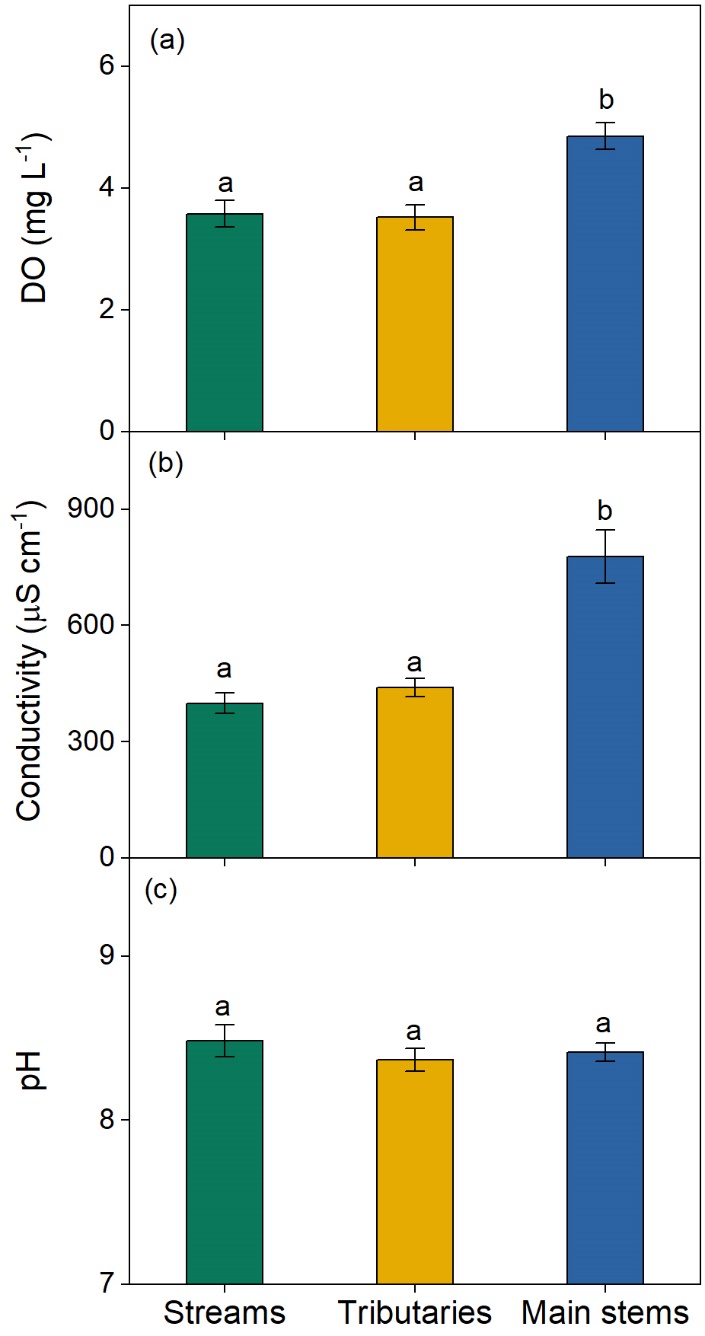
**Fig. S3** Changes in water properties along the streams, tributaries and main stems of the Yangtze River Source Region. DO, dissolved oxygen. Different lowercase letters indicate significant differences in environmental parameters among the different stream orders (LSD test, *P* < 0.05).

**References**

Irvine, K. N., J. E. Richey, G. W. Holtgrieve, J. Sarkkula, and M. Sampson. 2011. “Spatial and Temporal Variability of Turbidity, Dissolved Oxygen, Conductivity, Temperature, and Fluorescence in the Lower Mekong River-Tonle Sap System Identified Using Continuous Monitoring.” *International Journal of River Basin Management* 9: 151-168.

Jiménez, A., J. Aroba, M. L. de la Torre, J. M. Andujar, and J. A. Grande. 2009. “Model of Behavior of Conductivity Versus pH in Acid Mine Drainage Water, Based on Fuzzy Logic and Data Mining Techniques.” *Journal of Hydroinformatics* 11: 147-153.

Li, Y., X. Li, C. Xu, and X. Tang. 2023. “Dissolved Oxygen Prediction Model for the Yangtze River Estuary Basin Using IPSO-LSSVM.” *Water* 15: 2206.

Liu, G., W. He, and S. Cai. 2020. “Seasonal Variation of Dissolved Oxygen in the Southeast of the Pearl River Estuary.” *Water* 12: 2475.

Liang, X., Z. Jian, Z. Tan, et al. 2024. “Dissolved Oxygen Concentration Prediction in the Pearl River Estuary with Deep Learning for Driving Factors Identification: Temperature, pH, Conductivity, and Ammonia Nitrogen.” *Water* 16: 3090.

Zhang, X., L. Liu, X. Chen, Y. Gao, S. Xie, J. Mi. 2021. “GLC_FCS30: Global Land-cover Product with Fine Classification System at 30 m Using Time-series Landsat Imagery.” *Earth System Science Data* 13: 2753-2776.
